# Enzymatic Debridement of Deep Thermal Burns in the Russian Federation: First Experience

**DOI:** 10.3390/life13020488

**Published:** 2023-02-10

**Authors:** Andrey A. Alekseev, Natalia B. Malyutina, Alexander E. Bobrovnikov, Yaron Shoham

**Affiliations:** 1 National Medical Research Center of Surgery Named after A. Vishnevsky of the Ministry of Health of the Russian Federation, 115093 Moscow, Russia; 2 Russian Medical Academy for Continuing Professional Education of the Ministry of Health of the Russian Federation, 123995 Moscow, Russia; 3 Plastic Surgery Department and Burn Unit, Soroka University Medical Center, Beer Sheba 84101, Israel; 4 Faculty of Health Sciences, Ben Gurion University of the Negev, Beer Sheba 84105, Israel

**Keywords:** deep thermal burns, mixed depth burns, NexoBrid, enzymatic debridement, spontaneous healing

## Abstract

Since its approval in Europe a decade ago, NexoBrid^®^ enzymatic debridement of deep thermal burns has been gaining acceptance as standard practice around the world. The purpose of this study is to report the first experience with NexoBrid^®^ in the Russian Federation. During 2019–2020, we conducted a post-registration clinical study assessing the safety and treatment results of NexoBrid^®^ enzymatic debridement. The study involved 15 adult patients suffering from deep thermal burns over an area ≤15% of their total body surface area. Patients were treated with NexoBrid^®^ within 3 days of injury, followed by spontaneous or surgical wound closure. Complete eschar removal was achieved in twelve patients, 80% eschar removal in two patients, and 70% in one patient. Complete spontaneous epithelialization of wounds was achieved in 12 patients (80%) within 18 ± 1.9 days after the start of treatment. We did not witness pathological scarring during follow-up, and there were no significant safety issues throughout the study. Early use of NexoBrid^®^ resulted in rapid, effective, and safe eschar removal with good results and sufficient preservation of viable dermis to allow for spontaneous healing in 80% of patients. These results demonstrate the ability to minimize surgical intervention and hopefully lead to better long-term scarring results.

## 1. Introduction

Thermal damage to the skin still represents a challenge in excisional and reconstructive surgery. Particularly challenging are mixed-depth burns, where areas of deep eschar are combined with more superficial areas. Quinby et al. referred to these burns as “mosaic” [1]. These wounds may have the potential for slow autolytic debridement and subsequent healing. However, the lengthy presence of the eschar increases the risk for development of infections that may endanger the patient’s life and lead to the formation of significant post-burn scarring [2,3,4,5].

Therefore, it is crucial to complete the removal of the eschar as early as possible after its formation [2,6,7]. Currently, it is common medical practice to remove the eschar using surgical, chemical, and enzymatic eschar removal methods [8]. Surgical debridement is usually followed by either simultaneous or delayed skin-grafting [9]. The main advantages of this method are the early and efficient removal of necrotic tissues and, consequently, the reduction of infections and their complications, the reduction of pathological scarring, and the reduction of treatment time and costs. The disadvantages of surgical debridement are significant blood loss; the need for anesthesia; damage to healthy tissues due to unnecessary removal of viable tissue during the process or, on the contrary, insufficient excision of damaged tissues; and pain in the post-surgery period [2,6,7,8,10,11]. In the Russian Federation, chemical debridement (necrolysis) is normally carried out with the use of 40% salicylic ointment, which reduces inflammation under dried eschar. As a result, partial dissolution of tissue occurs at the border between viable and dead tissue [12]. The disadvantages of this method are not only pain and possible worsening of the patient’s condition, but also the lengthy duration of the procedure, which is explained by the need for preliminary, obligatory drying of eschar with subsequent application of salicylic ointment for 48 h. There are also enzymatic methods of debridement that allow the removal of eschar using proteolytic enzymes such as Travase, Collagenase, and Varidase. However, their clinical use is very time-consuming, involving multiple applications of enzymes to the eschar for 5–7 days, and the result is not always satisfactory [8,13,14,15].

Since its approval in Europe a decade ago [16], a technique for enzymatic debridement using bromelain gel (NexoBrid^®^) has been gradually gaining acceptance around the world [17,18]. NexoBrid^®^ was developed by MediWound Ltd. (Yavne, Israel) and contains a mixture of proteolytic enzymes enriched in Bromelain. The mechanism of action is based on the ability of the proteolytic enzymes to rapidly dissolve non-viable tissues while not harming viable tissues during the process [19]. NexoBrid^®^ has been shown to be effective and selective in removing eschar in deep thermal burns [20,21,22,23].

Following approval in other regions around the world, NexoBrid^®^ was approved for use in the Russian Federation in 2018. The aim of this study was to assess the safety and efficacy of initial experience in the Russian Federation using NexoBrid^®^ enzymatic debridement in patients with mixed-depth thermal burns. To the best of our knowledge, this is the first report of such an experience with NexoBrid^®^ in the Russian Federation.

## 2. Materials and Methods

During 2019–2020, the State Budgetary Healthcare Institution “F.I. Inozemtsev Municipal Clinical Hospital” in Moscow conducted an observational post-registration clinical study assessing the results of enzymatic burn wound debridement using NexoBrid^®^. The clinical study was conducted in accordance with the ICH GCP guidelines, the ethical principles stated in the WMA Declaration of Helsinki, the EU Clinical Trials Directive 2001/20/EC, and the requirements of the Russian legislation. The clinical study was approved by the Local Committee for Ethics (Protocol No. 2 as of 19 June 2019).

### 2.1. Inclusion Criteria

Adult patients suffering from mixed-depth partial-thickness thermal burns; covering no more than 15% of the total body surface area (TBSA); who experienced burn trauma no more than 3 days before entering the clinical study; and women who tested negative for pregnancy. 

### 2.2. Exclusion Criteria

Patients were excluded in case of burns involving more than 15% TBSA; patients with electrical and chemical burns, as well as burns contaminated with radioactive or other hazardous substances; patients with inhalation injury; patients with penetrating wounds or damage caused by a combination of factors; patients with perineum and genital burns; patients with impaired blood clotting according to coagulation tests (increased risk of bleeding) and hemorrhagic diathesis; patients with established allergic reactions or other types of intolerance to active and auxiliary substances contained in the study drug, as well as patients with a history of severe immediate hypersensitivity reactions (including anaphylaxis) to drugs containing bromelain and papain, including allergic reactions to fruits (pineapple, papaya, etc.), latex, bee venom and olive tree pollen; patients with a history of malignant tumors and severe, decompensated or unstable systemic diseases (any disease or condition that threatens the patient’s life or worsens the patient’s prognosis, and also makes it impossible for the patient to participate in the study; and patients who participated in other clinical studies within 3 months prior to enrollment in this study. 

### 2.3. Pre-Enrollment Assessment

All patients were admitted to the burn center by ambulance and had indications for enzymatic debridement. The procedure was carried out after signing an informed consent, physical examination, assessment of condition severity, describing the burn wound, pain assessment by VAS, assessment of vital signs, instrumental and laboratory research in accordance with the standard of medical care, laboratory and instrumental examinations in accordance with medical standards of care, registration of concomitant therapy, checking the inclusion/non-inclusion criteria within a period not exceeding 3 days from the time of receiving burn injuries.

### 2.4. Enzymatic Debridement

**Figure 1:**
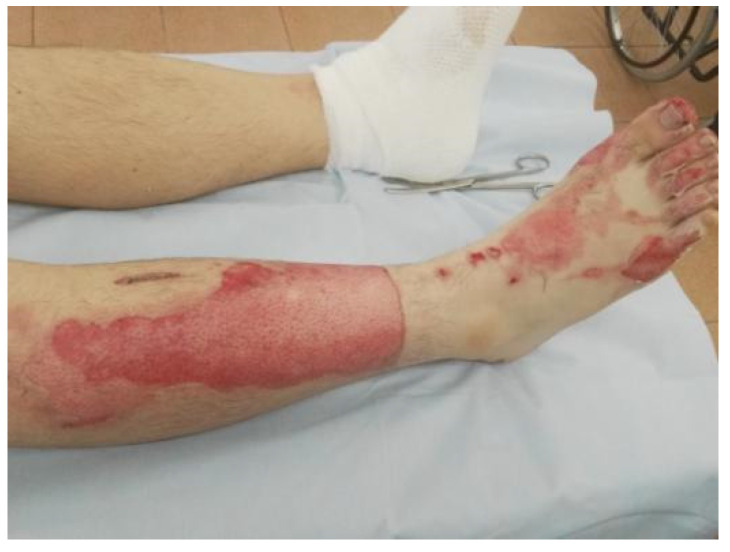
Mixed-depth, partial-thickness flame burns on the right lower extremity, 45 h after trauma.

**Figure 2:**
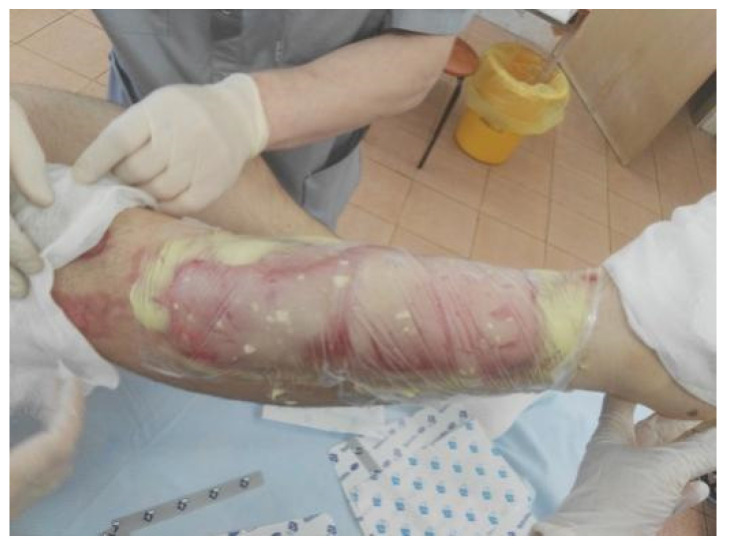
NexoBrid^®^ gel is applied. The wound is covered with an occlusive film dressing.

**Figure 3:**
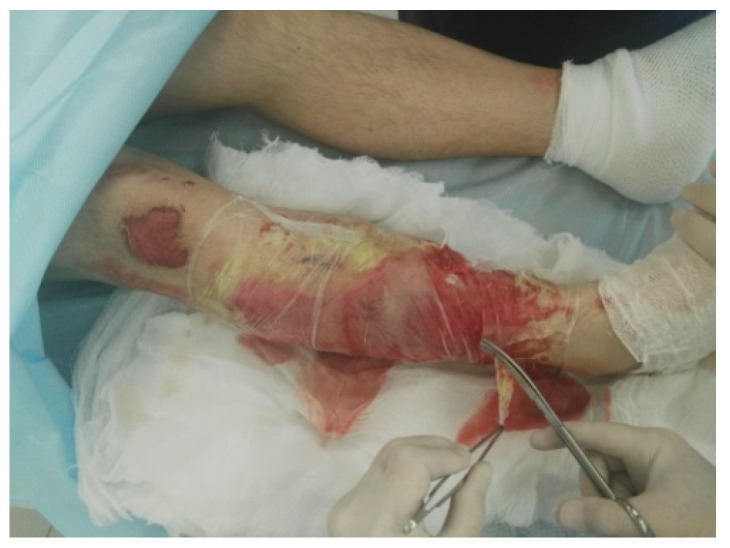
Removal of the occlusive dressing, gel, and wound cleaning 4 h later.

**Figure 4:**
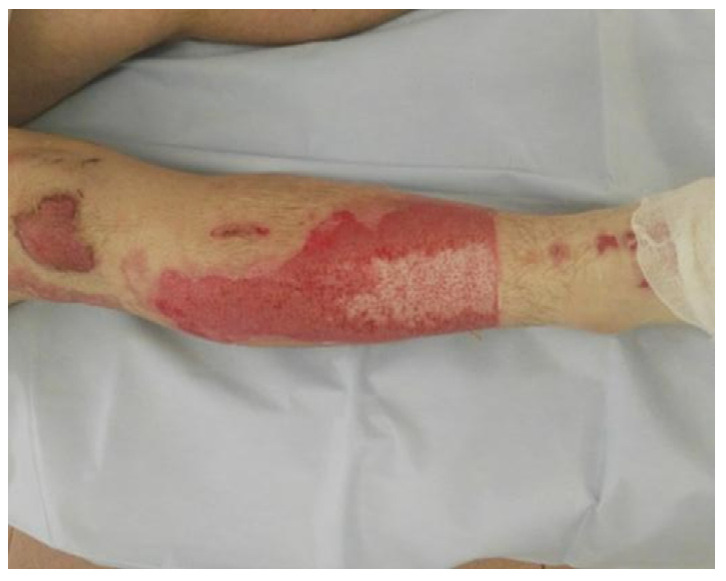
The wound is clean.

**Figure 5:**
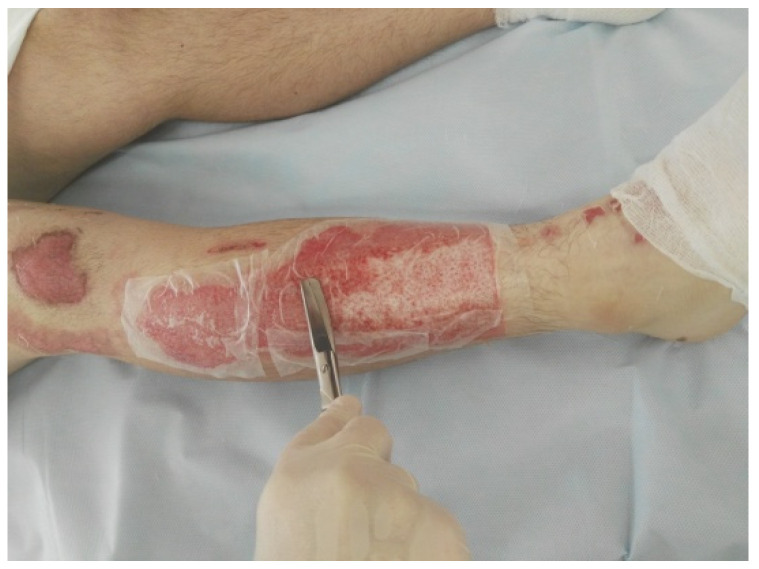
Application of an alloplastic skin substitute.

**Figure 6:**
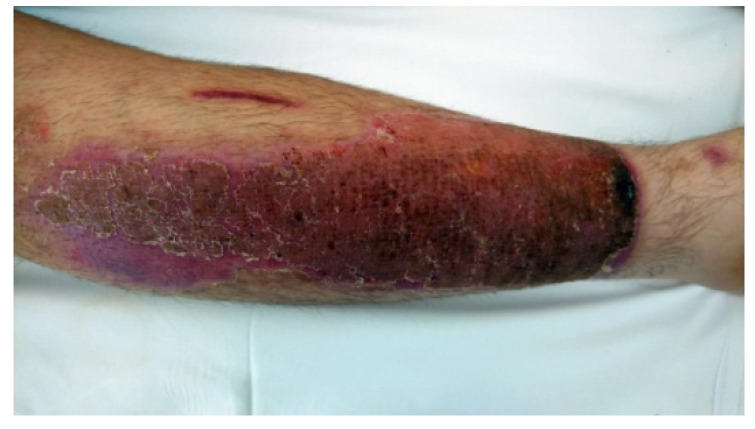
Full epithelialization 9 days later.

Enzymatic debridement was performed using NexoBrid^®^ (Mediwound Ltd., Yavne, Israel). The procedure was performed in accordance with the instructions for use. Initially, an antibacterial soaking with a solution of chlorhexidine bigluconate 0.05% was applied to the wound surface 2 h before the procedure of enzymatic debridement. Then the burn wound was thoroughly cleared of keratin and exfoliated epidermis (Figure 1). Sterile paraffin or a hydrophobic-based ointment (e.g., Methyluracil) was applied to the area around the wound where the eschar was to be removed as an adhesive barrier to prevent the leaking of NexoBrid^®^. The preparation of the gel was carried out under aseptic conditions immediately before use. The contents of the vial with the lyophilized enzyme powder were carefully transferred to the gel vial and mixed for 1–2 min using a sterile spatula until a homogeneous, light brown gel was formed. The gel was applied within 15 min of preparation. The freshly prepared gel was applied in a 1.5–3 mm layer on the wound that had previously been cleaned and moistened with a sterile 0.9% sodium chloride solution. Next, the wound was covered with an occlusive dressing pressed against the adhesive barrier and the NexoBrid^®^ gel (with no air pockets under the dressing), which was then fixed with a loose bandage (Figure 2). The bandage was left in place for 4 h. During this period, the patient was recommended bed rest to ensure maximum contact of the gel with the wound surface. Four hours after the application, the dressing was removed (Figure 3). The dissolved eschar was removed using sterile instruments (e.g., spatula, brush) and gauze to the point where a pinkish surface with pin-point bleeding or whitish tissue (reticular dermis) became clearly visible (Figure 4). After the eschar was removed, an antibacterial soaking was applied to the wound for 2 h. Two hours after the removal of NexoBrid^®^, burn wounds were examined, and the strategy for further local treatment was determined. At that time, conservative therapy was used in all patients, i.e., treatment with biological or synthetic (i.e., lyophilized pig skin, Suprathel^®^, or mesh atraumatic dressings) wound dressings (Figure 5). The total duration of the procedure from the application of the first antibacterial dressing to the application of a biological or synthetic wound dressing averaged 8.5 h. Additional evaluation of treatment efficacy and the subsequent need for skin autografting versus treatment towards spontaneous healing was carried out on days 4–5 after enzymatic debridement (Figure 6). The frequency of dressings at the beginning of treatment was 2–3 times a week and decreased as the area of the wounds decreased. 

### 2.5. Pain Control

An important aspect of enzymatic debridement is adequate pain relief. Pain intensity was assessed by the Visual Analog Scale (VAS), rating pain from 0 (no pain) to 10 (worst pain). Non-narcotic analgesics (e.g., acetaminophen, NSAIDs) were used in all patients. Additionally, in most patients, removal of the gel and wound debris also required intravenous anesthesia using agents such as fentanyl, propofol, and ketamine. 

### 2.6. Endpoints

The primary endpoint was the percentage of patients in whom removal of ≥90% of the eschar was achieved. Secondary endpoints included the percentage of patients requiring skin grafting; the time to complete wound closure (in days); and the assessment of the functional and aesthetic results. Eschar removal and the need for grafting were assessed by the clinical judgment of surgeons experienced in burn care. Complete wound closure was assessed as complete epithelialization without the need for further dressings. Signs of hypertrophic scarring and limitations to range of motion were assessed by the investigators 1 month after wound closure.

### 2.7. Statistical Analysis

Data processing was carried out using the statistical packages Statistica 10.0 and SPSS Statistics 21 (IBM^®^, New York, NY, USA).

## 3. Results

Fifteen patients were enrolled and treated during 2019–2020, including three women and twelve men aged 25–67 (mean 37.3 ± 11.6) years old. The burn area at admission ranged from 3–15% (mean 9.5 ± 4.1%) TBSA; the area of mixed-depth partial-thickness burns on which enzymatic debridement was to be performed ranged from 1–3% (mean 2.0 ± 0.6%) TBSA. Enzymatic debridement locations were the upper extremity in two patients, the lower extremity in five patients, the trunk in two patients, the hand in five patients, the foot in one patient, and the neck in one patient. Concomitant diseases at the time of enrollment in the study were found in four patients: bronchial asthma in two patients, coronary heart disease in one patient, and hypertension in one patient. The main patient and wound characteristics are shown in Table 1.

Enzymatic debridement was initiated 32–66 (mean 49.9 ± 1.2) hours after injury. During the preparation of a burn wound for enzymatic debridement, as well as during the procedure and after removing the bandage, a dynamic assessment of pain intensity was performed using a visual analogue scale (VAS). The VAS pain score averaged 2.7 ± 0.3 points before the application, 7.5 ± 0.3 during the application (before the addition of intravenous anesthesia), and 5.8 ± 1.2 after the removal of the bandage. Evaluation of enzymatic debridement efficacy was carried out after the removal of the occlusive dressing. Complete removal of the burn eschar was achieved in twelve out of the fifteen patients in the study group, 80% of the eschar was removed in two patients, and 70% in one patient. It was observed that the effectiveness was reduced when used on an uneven (convex or concave) wound surface due to “leaking” of the gel; in our study, incomplete removal of the eschar was noted in the areas of the shoulder, neck, and dorsum of the fingers.

After enzymatic debridement, in order to create optimal conditions for wound epithelialization, a temporary wound dressing was used: lyophilized pig skin in four patients, Suprathel^®^ in four patients (26.66%), and mesh atraumatic dressings in seven patients. There were no significant differences in the wound healing process, the timing of wound epithelialization, or the frequency of adverse events when using different types of coverages during the study. Complete spontaneous epithelialization of wounds was achieved in 12 patients. In three patients, the wounds healed partially, then they underwent skin autografting of the non-healed areas. Among the 12 patients with complete spontaneous epithelialization of the burn wound, the duration of wound healing was 8–30 (average 18 ± 1.9) days after the start of treatment.

Evaluation of functional and aesthetic results 1 month after wound closure was carried out in 14 (93.3%) patients. In 13 (92.8%) patients, the goals of treatment were achieved: complete restoration of the skin and the function of the affected organ without limitations in range of motion, as well as the absence of hypertrophic scarring. In one patient, due to the early termination of observation, it was impossible to make any conclusions about the final result of treatment.

During the course of the study, safety assessments indicated that pain was an adverse event associated with the application of NexoBrid^®^. We witnessed higher pain levels than we anticipated after the initiation of enzymatic debridement, which we addressed with the addition of intravenous anesthesia.

## 4. Discussion

In recent years, due to the widespread introduction of minimally invasive technologies, surgical treatment of burns has given way to alternative and less aggressive interventions, in particular enzymatic debridement [17,18,22,23]. The method of enzymatic debridement using NexoBrid^®^ was studied elaborately for decades [24], and since 2012, after initially being approved for use in Europe [16], it has been gaining acceptance as standard practice in many countries around the world [17,18,25,26,27,28,29]. Early use of this bromelain-based gel has been reported to result in rapid, selective, efficient, and safe (compared to standard treatment methods) eschar removal without damaging the uninjured dermis. At the same time, a decrease in the number of surgical interventions, blood loss, and even mortality rates have also been reported [19,20,22,30,31].

According to published data, NexoBrid^®^ successfully and selectively debrides mixed-depth burn wounds and creates an enabling environment for spontaneous epithelialization. In many cases, patients do not need skin auto-transplantation, and wounds heal spontaneously under wound dressings or temporary skin substitutes [20]. Our initial experience demonstrated in this trial is in line with these reports. Twelve of our fifteen patients achieved complete eschar removal as a result of the NexoBrid^®^ application. We believe that the three patients who achieved only partial eschar removal (70–80%) were the result of a technical issue where the contact between the NexoBrid^®^ and the wound bed was incomplete due to uneven anatomical surfaces. There is a learning curve associated with the use of NexoBrid^®^, and we believe learning to overcome such issues is part of it [32,33,34].

Another issue that pertains to the learning curve is that of the post-NexoBrid^®^ wound bed assessment and subsequent wound management. We chose to concentrate specifically on mixed-depth, partial-thickness burns in order to benefit the most from the selective enzymatic debridement and to assess the implications of unharming viable dermis during debridement on the potential for spontaneous healing. Due to this, we initially decided to wait for spontaneous healing and subsequently autograft only those areas where signs of healing were not seen after 2 weeks. We believe that the fact that we had to autograft only three of our patients and the other twelve healed spontaneously within an average of 18 days reflects the selectivity of the enzymatic debridement process, which is again in line with the previous reports mentioned above. We chose to treat relatively small areas with NexoBrid^®^ in this initial experience (up to 3% TBSA) in order to gain experience and alleviate the learning curve.

In perspective, a study reporting results after surgical dermabrasion seems comparable to enzymatic debridement in some respects. When using synthetic skin substitutes after dermabrasion, the duration of epithelialization for mixed-depth partial-thickness burns averaged 15 days after injury, and complete epithelialization of wounds was achieved in 90% of patients [35]. The duration, labor intensity, and cost of the dermabrasion procedure are lower. Perhaps better standardization of burn depth and more experience with enzymatic debridement are needed to be able to accurately compare these results with the results of our current study.

We applied NexoBrid^®^ in this study within 66 h of injury. This is in line with the European consensus guidelines that state that application after 72 h of injury may be performed in some cases, but only after a prolonged pre-NexoBrid^®^ soaking period [17,18]. Delayed application of NexoBrid^®^ has also been reported by Waldner et al. [36]; however, in this initial experience, we chose to treat within the first 72 h.

There are published positive healing results with a wide array of post-NexoBrid^®^ dressings, e.g., Suprathel, Biobrane, saline soaks, and silver sulfadiazine [18,32,34,37,38,39]. However, as of today, no single agent has been shown to be superior to others for treating the post-enzymatic debridement wound bed. In our study, we treated the post-NexoBrid^®^ debrided wound bed with either a biological dressing (lyophilized pig skin) or synthetic dressings (Suprathel^®^ or mesh atraumatic dressing) in attempts to assess whether any of these dressings would prove to facilitate spontaneous healing better than the others. While it is true that two of the patients who later necessitated autografting were treated with mesh atraumatic dressings after NexoBrid^®^, we do not think the dressing type was responsible for this difference. It is more likely that the incomplete debridement in these cases is the reason for the need for subsequent autografting. Therefore, we cannot deduce the superiority or inferiority of any of these dressings at this stage. 

Regarding the timing for autografting, the European consensus guidelines recommend waiting at least 2 days between enzymatic debridement and grafting [17,18]. The reason for this may originate from the findings of Di Lonardo et al., who reported that post-NexoBrid^®^ debrided wound bed biopsies in patients demonstrated that the topmost layer of the wound bed contained partially viable skin annexes and resembled a dermal matrix [40]. Therefore, waiting several days for this layer to regain full viability is recommended before autografting. In our study, we had initially anticipated waiting 4–5 days before autografting for this reason. However, after assessing the wounds at this stage, we chose to wait longer as we were not sure about the exact healing potential of some of the wounds. This too can be associated with the early stages of the learning curve. Eventually, we autografted certain areas in three of our patients after seeing insufficient signs of healing after 2 weeks.

Effective pain management may also be considered part of the learning curve associated with NexoBrid^®^ [41]. The fact that a topical gel application may cause significant pain levels is not trivial and may be surprising at first. However, witnessing that this gel is capable of achieving complete eschar removal within 4 h helps understand this issue. Indeed, we witnessed elevated pain scores in our patients prior to administering intravenous anesthesia and therefore recommend initiating appropriate anesthesia earlier, in addition to premedication with analgesics. Other pain management modalities worth considering have been reported, including nerve blocks [42,43]. 

In this initial experience with NexoBrid^®^, we followed up on the patients for only 1 month after wound closure. During this timeframe, we did not witness any hypertrophic scarring or limitations in range of motion. While this experience is rather small and short, it is in line with the results of a recently completed phase III multicenter randomized controlled trial, where NexoBrid^®^ treated patients had significantly better Modified Vancouver Scar Scale (MVSS) scores after 1 year of follow-up when compared to those treated with standard eschar removal procedures [44]. This is also in line with other previous reports of positive scarring outcomes in NexoBrid^®^-treated patients [43,45,46]. We intend to perform follow-ups longer than 1 month in future studies and recommend this to others as well.

We believe that the results of our initial experience in Russia demonstrate the safety and efficacy of NexoBrid^®^ and are in line with previously published studies around the world. Currently in Russia, NexoBrid^®^ is registered for use in adult patients with a burn area of no more than 15% of the body surface area. Treatment of children, the elderly, large burns >15% TBSA, and patients with a wide range of concomitant diseases is currently considered off-label. Ironically, these are the categories of patients in which the use of minimally invasive technologies is probably most justified. Despite the labeling restrictions, there are a significant number of publications on the use of NexoBrid^®^ in severely burned patients, including patients with burns over 60% TBSA, children, and other off-label categories of patients [18,30,47,48,49,50]. We believe these reports may reflect the additional potential benefits available with more widespread use of NexoBrid^®^; however, additional studies are needed to better assess these indications. An additional important issue to further explore is that of pain control.

We believe our study has two main strengths. First, to the best of our knowledge, this is the first report of the use of NexoBrid^®^ in the Russian Federation. Our initial experience may thus help pave the way for others in Russia to explore the use of enzymatic debridement. We believe the second strength of our study is that we chose to concentrate on mixed-depth partial-thickness burns, where the effect of the selectivity of NexoBrid^®^ may be more pronounced. By being able to salvage the viable dermal elements in mixed-depth partial-thickness burns, one can expect a shift towards a higher incidence of spontaneous epithelialization as opposed to a higher need for skin autografting with the use of the traditional surgical debridement technique. Indeed, we saw a high incidence of spontaneous healing in our patients.

Our study has several limitations. First, this study was observational, without a control group. Thus, it is difficult to numerically quantify the effects of NexoBrid^®^ efficacy and selectivity, such as the effects on the need for performing surgical debridement to achieve complete eschar removal and the need for skin autografting. Additionally, in this study, we were not able to assess the long-term effects of treatment as only a one month follow-up was performed. We were also unable to identify any superior post NexoBrid^®^ wound dressing. We recommend future trials include a control group, perform a longer follow-up, and attempt to identify dressings that are most effective in treating the post-enzymatic debridement wound bed.

## 5. Conclusions

Based on our initial experience, the use of enzymatic debridement in the treatment of patients with mixed-depth partial-thickness burns allows us to quickly and effectively clean the wound from necrotic tissues, create an enabling environment for the facilitation of spontaneous healing from the remaining skin derivatives, reduce the need for surgery, and hopefully improve long-term results. We find it important to additionally state that we believe the procedure of enzymatic debridement is quite costly financially and includes a learning curve in terms of time and resources. Therefore, we believe the introduction of this technique in the Russian Federation should be carried out in burn centers with sufficient personnel and material support, according to personal reasonable indications. We recommend future studies in Russia and other regions focus on larger cohorts than our initial experience included, including treatment of larger areas within the approved label with longer follow-up periods. We believe this may improve the possibility of correctly reporting the outcomes of enzymatic debridement.

## Figures and Tables

**Table 1:** Patient and wound characteristics.

No.	Sex (M/F)	Age (Years)	Burn Area (%TBSA)	NexoBrid Application Area Depth and %TBSA		Eschar Removal (%)	Post NexoBrid Dressing Used	Skin Autografting Performed
1	M	26	12	Mixed ^1^	3	100	Suprathel	No
2	M	35	15	Mixed ^1^	3	100	Lyophilized pig skin	No
3	M	31	14	Mixed ^1^	3	100	Mesh atraumatic dressing	No
4	M	36	3	Mixed ^1^	2	100	Mesh atraumatic dressing	No
5	f	27	11	Mixed ^1^	1.5	100	Suprathel	No
6	M	56	10	Mixed ^1^	2	100	Suprathel	No
7	M	52	5	Mixed ^1^	2	70	Mesh atraumatic dressing	Yes
8	M	29	10	Mixed ^1^	2	100	Suprathel	No
9	M	34	14	Mixed ^1^	2	100	Mesh atraumatic dressing	No
10	M	25	11	Mixed ^1^	2	100	Mesh atraumatic dressing	No
11	M	67	5	Mixed ^1^	1	80	Mesh atraumatic dressing	Yes
12	M	36	15	Mixed ^1^	2	100	Mesh atraumatic dressing	No
13	f	35	8	Mixed ^1^	2	100	Lyophilized pig skin	No
14	M	30	5	Mixed ^1^	1	100	Lyophilized pig skin	No
15	M	40	4	Mixed ^1^	1	80	Lyophilized pig skin	Yes

^1^ Mixed-depth partial-thickness burn.

## Data Availability

The data presented in this study are available on request from the corresponding author. The data are not publicly available due to policy restrictions.
